# Low-Density Lipoprotein Receptor-Related Protein 8 at the Crossroad between Cancer and Neurodegeneration

**DOI:** 10.3390/ijms23168921

**Published:** 2022-08-10

**Authors:** Daniela Passarella, Silvia Ciampi, Valentina Di Liberto, Mariachiara Zuccarini, Maurizio Ronci, Alessandro Medoro, Emanuele Foderà, Monica Frinchi, Donatella Mignogna, Claudio Russo, Carola Porcile

**Affiliations:** 1 Department of Medicine and Health Sciences “V. Tiberio”, University of Molise, 86100 Campobasso, Italy; 2 Department of Experimental Biomedicine and Clinical Neurosciences, University of Palermo, 90133 Palermo, Italy; 3 Department of Medical Oral and Biotechnological Sciences, University of Chieti-Pescara, 66100 Chieti, Italy; 4 Department of Pharmacy, University of Chieti-Pescara, 66100 Chieti, Italy

**Keywords:** LRP8, cancer, Alzheimer’s disease, apolipoprotein, LDL receptor family

## Abstract

The low-density-lipoprotein receptors represent a family of pleiotropic cell surface receptors involved in lipid homeostasis, cell migration, proliferation and differentiation. The family shares common structural features but also has significant differences mainly due to tissue-specific interactors and to peculiar proteolytic processing. Among the receptors in the family, recent studies place low-density lipoprotein receptor-related protein 8 (LRP8) at the center of both neurodegenerative and cancer-related pathways. From one side, its overexpression has been highlighted in many types of cancer including breast, gastric, prostate, lung and melanoma; from the other side, LRP8 has a potential role in neurodegeneration as apolipoprotein E (ApoE) and reelin receptor, which are, respectively, the major risk factor for developing Alzheimer’s disease (AD) and the main driver of neuronal migration, and as a γ-secretase substrate, the main enzyme responsible for amyloid formation in AD. The present review analyzes the contributions of LDL receptors, specifically of LRP8, in both cancer and neurodegeneration, pointing out that depending on various interactions and peculiar processing, the receptor can contribute to both proliferative and neurodegenerative processes.

## 1. Introduction

Cancer and neurodegeneration represent two age-related conditions that share common biochemical features, and it is not clear what triggers can lead to one or the other condition through apparently common routes and pathways [1,2,3]. It is well-known that the activation and deregulation of the cell cycle may lead to cell death in neurons and to uncontrolled proliferation in malignant cells; however, there is still a gap in the understanding of the precise molecular processes involved in both disorders; unraveling them represents an important opportunity to identify prognostic markers and to achieve therapeutic progress in both pathological conditions.

The low-density-lipoprotein receptors (LDLRs) represent a family of cell surface receptors originally linked to lipoprotein and lipid trafficking and, more recently, considered pivotal regulators of signal transduction pathways for cell proliferation, migration and differentiation [4]. Recent growing evidence has also highlighted their associations with neurodegenerative disorders and cancer due to the modulation of specific molecular pathways related to neuronal cell death and, in parallel, to cell proliferation, cell cycle activation and metastatic invasion [5,6,7,8,9].

## 2. LDLR Family

Listed in the order of their discovery, the main members of the LDLR family are: LDLR; LDLR-related protein (LRP, LRP1); Megalin (LRP-2, originally called gp330); very low-density lipoprotein (VLDL) receptor (VLDLR; in chicken termed LR8); LR11 (also named SorLA); apolipoprotein E (ApoE) receptor type 2 (LRP8, LRP-8) [4]; LRP-3, -4, -5, and -6, and LR32 (also termed LRP1B).

**Figure 1:**
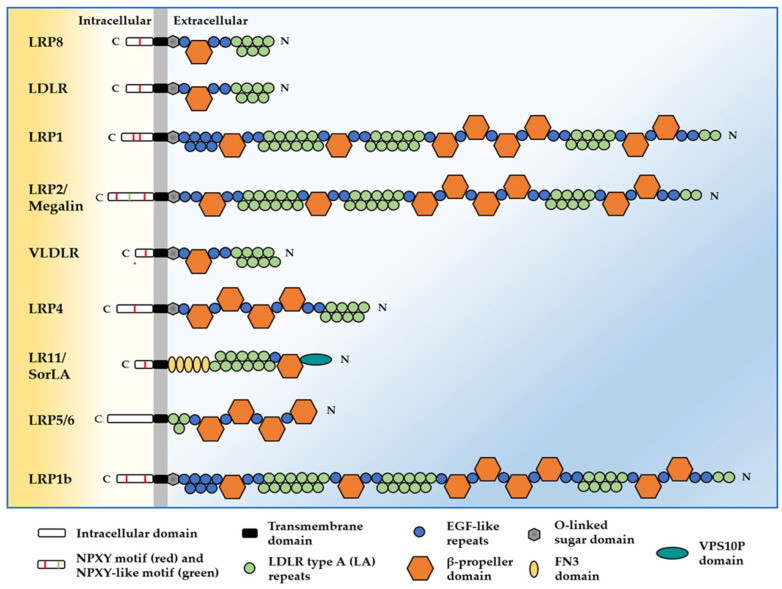
The domain structure of the LDL family members: differences and similarities. LDLR, VLDLR, and LRP8 are made of seven LA repeats in the ligand-binding domain, three EGF-like domains, and one YWTD β-propeller. LRP1 and LRP2 have the largest extracellular domains, each with eight YWTD β-propellers spaced by EGF and LA repeats. LRP4, LRP5 and LRP6 carry high sequence homology to a region within LRP1 with YWTD β-repeats going three through six. SorLA has two additional domains: the FNIII domain and the VPS10 domain. Regarding the NPxY motif, VLDLR, LRP8 and LR11/SorLA-1A have a single copy, whereas LRP1 and LRP1B contain two copies and LRP2/Megalin three copies. No NPXY is present in LRP5 and LRP6. Domains are not drawn to scale.

The structure of the LDL receptors, described in Figure 1, contains five common functional regions: a N-terminal ligand-binding domain with a variable number of LDLR type A (LA) repeats, an epidermal growth factor precursor homology (EGFP) domain composed of EGF-repeats (cysteine-rich class B repeats) and a YWTD (Tyr–Trp–Thr–Asp)/β-propeller domain, an O-linked sugar domain, a transmembrane domain and a cytoplasmic domain containing at least one NPxY (Asn-Pro-Xaa-Tyr) motif [10,11,12]. SorLA has two additional domains: the fibronectin type III (FNIII) domain and the vacuolar protein sorting 10 (VPS10) homology domain. In addition to the LDLR members, the NPXY motif is found in a subset of cell surface proteins well-known to be involved in cell proliferation and cancer including APP [13], growth factor receptors [14] and integrins [15].

The LDLR family participates in a wide range of physiological processes in different organs, tissues and cell types [4]. They are mainly known for binding and internalizing several extracellular ligands, including lipoproteins, exotoxins and lipid-carrier complexes, as well as for mediating signaling responses as a result of changes in the extracellular environment. The presence of the NPxY motif in the cytoplasmic domain represents an intracellular binding site for many adaptor proteins such as the mammalian Disabled-l (mDab-1), mDab-2, FE65, JNK-interacting protein JIP-1 and JIP-2, PSD-95, CAPON, and SEMCAP-1 [16,17,18]; many of these contain in their structure at least one of the three frequently found interaction domains such as phosphotyrosine binding (PTB), PDZ or SH2-SH3 domains or ankyrin repeats.

A functional peculiarity of the LDL receptors, especially LRP8, is their double functional role in neurodegeneration and cancer, the main focus of this review, as summarized in Table 1.

### 2.1. LDLR

The low-density lipoprotein receptor (LDLR) is the prototype of a classical endocytosis receptor that mediates the uptake of ligands and is broadly expressed on multiple cell types in various tissues. In the CNS, the LDLR plays an important role in regulating the homeostasis of cholesterol in the blood and also within tissues and cells [19]. The main well-known LDLR ligand is represented by apolipoprotein E (ApoE), a 34 kDa soluble protein that is a main component of lipoproteins in plasma and a secreted glycoprotein of 34 kDa that acts as a cholesterol carrier and signaling molecule. Through its interaction with LDLR, ApoE plays a critical role in peripheral cholesterol metabolism [20] and also in cholesterol transport into the central nervous system (CNS) [21,22]. LDLR is the only member of this receptor family to demonstrate an apoE isoform-specific binding affinity (E4 > E3 >> E2). Evidence suggests that a functional interaction between ApoE and LDLR influences the risk of cardiovascular disease (CVD) and AD [23]. The apoE–LDLR interaction may also play a more direct role through the modulation of amyloid clearance and/or deposition. Several intriguing studies have shed some light on the potential pathophysiological pathways by which LDLR may contribute to AD. In cell culture, overexpression of the amyloid precursor protein (APP) led to the increased expression of LDLR and altered receptor localization [24]. Varying results in amyloid deposition have been shown in AD mouse models lacking the LDLR, with one study showing that amyloid deposition was enhanced on a LDLR null background [25], while in another study, LDLR deficiency had no effect on brain amyloid-β peptides (Aβ) levels [26].

Kim et al., showed that LDLR over-expression reduced brain ApoE levels and amyloid β aggregation [27], while further work from the same laboratory showed that LDLR over-expression in the brain increased the rate of brain-to-blood clearance of both exogenously administered and endogenous Aβ [28]. These studies, in which LDLR over-expression increases Aβ clearance, point to the upregulation of LDLR in the brain as a potential therapeutic strategy for AD.

Considering the vital role of the LDL/LDLR routine in regulating blood and intracellular cholesterol homeostasis, several studies have focused on the function of LDLR in cancer progression. Chen et al., have demonstrated that the reduced expression of LDLR in hepatocellular carcinoma cells impairs LDL uptake but promotes proliferation and metastasis in vitro and in vivo by activating MEK/ERK [29].

### 2.2. LRP1

LRP1 represents a key receptor in maintaining lipid metabolism. The expression of LRP1 is ubiquitous, and its upregulation has been reported in numerous human diseases. In addition to its function as a scavenger receptor for various ligands, LRP1 transduces multiple intracellular signal pathways including mitogen-activated protein kinase (MAPK), Akt, Rho and the integrin signaling pathway. LRP1 signaling plays an important role in the regulation of diverse cellular processes, such as cell proliferation, survival, motility, differentiation and transdifferentiation, and thus, it participates in the pathogenesis of organ dysfunction and injury [30]. LRP1 is widely expressed throughout the CNS, where it modulates neuronal survival, neurite outgrowth, regeneration and calcium signaling through different ligands and cell-specific mechanisms. Indeed, LRP1 recognizes and is involved in the endocytosis of more than 40 different ligands including ApoE, APP and Aβ [31,32,33]. Depending on its binding to different ApoE isoforms, LRP1 is involved in calcium-related cellular processes such as increasd resting calcium, calcium response to N-methyl-D-aspartate (NMDA) and neurotoxicity [34]. Moreover, LRP1 binding to lipoprotein particles containing ApoE 3/4 protects against apoptosis upon the activation of protein kinase Cδ (PKCδ) and the inactivation of glycogen synthase kinase-3β (GSK3β), with a greater effect due to lipoproteins with ApoE4 than those with ApoE 3 [35]. The majority of research examining LRP1 function in the brain has focused on APP trafficking [36,37], Aβ clearance from the brain parenchyma [38] and blood–brain barrier permeability [39]. Studies on knockout mouse models have shown that LRP1 deficiency is lethal early in development [40], whereas mice lacking LRP1 exhibit severe motor and behavioral abnormalities, hyperactivity, age-dependent dendritic spine degeneration, synapse loss, neuroinflammation, memory loss and eventual neurodegeneration [41,42]. In addition to its role in neuronal-related physiological processes, an additional role in cancer development has been associated with LRP1. In particular in human glioblastoma U87 cells, LRP1 promotes cellular migration and invasion by inducing the expression of the matrix metalloproteinase-2 (MMP-2) and MMP-9 [43]. Moreover, in U87 cells, Song et al., have proposed that phosphorylated ERK is a potential mediator of LRP1-regulated MMP expression. An additional piece to this puzzle has been provided by Fayard et al., suggesting that the serine protease inhibitor serpin PN-1 is a ligand of LRP1 that activates ERK and stimulates, in turn, MMP-9 expression in breast tumor cell lines [44]. Supporting this hypothesis, an analysis of 126 breast cancer patients revealed that those patients whose breast tumors had elevated PN-1 levels had significantly higher probabilities of developing lung metastasis, but not metastasis to other sites, on relapse. Finally, it has been found that in Her-2/neu and triple-negative breast cancer (TNBC), there is an increased expression of LRP1 that is linked to neoplastic aggressiveness due to high histological grade, elevated mitotic index, Ki67 > 20% and tumor recurrence, while in endometrial carcinoma (EC), the increased expression of LRP1 is associated with p53 alterations and p16 protein overexpression [45].

### 2.3. LRP2/Megalin

LRP2 is a multiligand receptor mostly localized in the epithelial cells of the renal tubules [46] and the choroid plexus [47], as well as in the lateral ventricles [48] and in different CNS cells ranging from oligodendrocytes, retinal ganglion cells, cerebellar granule neurons and astrocytes to hippocampal neurons [49]. In the CNS, LRP2 modulates neuronal survival and regeneration [50] as well as learning- and memory-related physiological mechanisms such as neurite outgrowth and synaptic plasticity [49]. LRP2 neurotrophic activity is performed through its binding with Transthyretin [51], while its neurite outgrowth function seems associated with metallothionines [52] and the APP/Fe65 pathway [53]. In the blood–brain barrier, LRP2 plays a central role in the clearance/entrance of many proteins from the brain/cerebrospinal fluid, including Aβ, insulin, insulin-like growth factor (IGF)-I and ApoE. Together with LRP8, LRP2 is also known to transport selenium, which is transported in the form of the selenocysteine-enriched selenoprotein P1 (Sepp1) to target tissues, where it constitutes the active center of glutathione peroxidase, thioredoxin reductase and deiodinases. In the kidney, proximal tubule epithelial cells highly express LRP2, indicating its physiological role in the reabsorption of Sepp1. This function is confirmed by studies on LRP2-mutant mice displaying urinary selenium loss, which correlates with Sepp1 excretion in their urine and reduced selenium and glutathione peroxidase activity in the brain [54].

LRP2 genetic alterations have also been associated with pathological conditions. In particular, some LRP2 mutations lead to the protein loss of function, underlying an autosomal recessive disorder, Donnai–Barrow syndrome, characterized by several CNS functional defects [55]. Other genetic studies have associated a polymorphism located in the LRP2 promoter with AD risk, regardless of the ApoE genotype [56], as well as some LRP2 germ-line polymorphisms with increased risk of prostate cancer recurrence [57]. Moreover, somatic LRP2 mutations have been identified in gastric cancer [58]. In support of LRP2’s role in cancer, Andersen et al., have reported that LRP2 is more frequently expressed among malignant melanoma samples compared with benign counterparts and that the proliferation and survival rates of cultured LRP2-expressing melanoma cells decrease upon the siRNA-mediated knockdown of LRP2 [7].

### 2.4. VLDLR

VLDLR is highly expressed in adipose tissue, heart and skeletal muscle and is mainly known for the following roles: uptake of VLDL-derived lipids, enhancement of lipoprotein lipase activity and mediation of the Reelin-related pathway, together with LRP8 [59]. Reelin binding to VLDLR/LRP8 receptors activates intracellular Src family kinases (SFKs), which in turn phosphorylate the adaptor protein Disabled-1 (Dab1) in specific tyrosine residues. Although the activation of the Reelin pathway through VLDLR and LRP8 is well-known to mediate the development of the central nervous system, studies have highlighted differences between the two receptors in terms of splicing, localization, interactors and trafficking, leading to unique functions separate from the overlapping ones. In particular, the Pafah1b complex mediates the downstream effects of VLDLR on neuronal migration, but it is not necessary for the function of LRP8 [60].

In addition to its roles in neuronal physiological processes, studies have highlighted the role of VLDLR in cancer. Indeed, Lei He et al., have shown that VLDLR promotes cell proliferation and migration in adenocarcinoma SGC7901 cells via beta-catenin/TCF signaling [8], and Lei He et al., have correlated VLDLR II expression with lymph node and distant metastasis in gastric and breast cancer patients [61].

### 2.5. LRP4

Although it is mainly expressed in bone, where it regulates bone formation by inhibiting sclerostin in Wnt1/β-catenin signaling [62], LRP4 is also found in astrocytes of the prefrontal cortex and hippocampus, regions particularly vulnerable to AD [63]. Studies of the genetic deletion of the Lrp4 gene in 5xFAD male mice, an AD mouse model, have shown increased levels of Aβ plaques and decreased neurotransmission and cognition, supporting a link between LRP4 loss and AD [64]. In the brain, LRP4 plays roles in synaptic transmission, long-term potentiation (LTP), cognitive function [65] and adult hippocampal neurogenesis [66]. Through its ligand agrin, LRP4 regulates the formation and maintenance of the neuromuscular junction [67,68]. In particular, the agrin–Lrp4 interaction increases its binding with MuSK, which is activated and thus activates the kinase that via Dok7 and rapsyn mediates AChR clustering [69]. In addition to AD, LRP4 has been associated with cancer. In papillary thyroid cancer, LRP4 has been reported to be significantly overexpressed in both tissues and related cell lines (TPC1, BCPAP and KTC-1) in comparison with controls, likely playing a role in proliferation, migration and invasion by inducing phosphoinositide 3-kinases (PI3K)/AKT mediated epithelial–mesenchimal transition (EMT) [70]. LRP4 overexpression has also been observed in gastric cancer tissues, where it correlates with malignant proliferating clinical features, while in gastric cancer cells (MKN45, MGC803, BGC823 and AGS), LRP4 activates the PI3K/AKT under regulation of miR-140-5p [71].

### 2.6. LR11/SorLA

SorLA is highly enriched in the brain. As LRP1, SorLA interacts with APP, influencing its trafficking and regulating Aβ levels [72,73]. Indeed, SorLA represents one of the major cellular players contributing to abnormal APP processing and enhanced Aβ formation. In particular, SorLA acts as a retention factor for APP in trans-Golgi compartments/trans-Golgi network, with GGA and PACS-1 adaptor proteins involved in protein transport to and from the trans-Golgi network, preventing the release of the precursor into regular processing pathways [74]. Interestingly, a peculiar alternative splicing of SORL1, induced by a non-coding RNA, is associated with the impaired processing of APP and Aβ formation [75]. A consistent loss of SorLA is observed in AD brains, particularly in vulnerable regions [76], and variants in the SORL1 gene encoding SorLA have been associated with AD risk, adding considerable support to the hypothesis that SorLA is genetically linked to AD pathogenesis [77]. However, SorLA represents another LDLR whose overexpression is linked to cancer, and in particular, it has been observed in HER2-driven cancer cell lines where the receptor regulates endosomal trafficking and oncogenic fitness of HER2 [78]. SorLA and its released soluble form are also strongly elevated in acute leukemia. Remarkably, the increased levels of soluble SorLA level were reduced in patients who achieved complete remission [79,80].

### 2.7. LRP5/6

LRP5 and LRP6 are two LDL receptors with amino acid sequences with 71% identity [81]. Their extracellular domains are responsible for binding Wnt ligands and their inhibitors, such as Dickkopf-related protein 1 (DKK1) and sclerostin [82]; indeed, LRP5 and LRP6 are the key players that the Wnt pathway together with the Frizzled receptors [83,84,85]. In particular, Wnt ligands signal through at least three different pathways: the canonical or Wnt/β-catenin pathway and the two noncanonical Wnt/JNK and Wnt/Ca2+ pathways. In the canonical pathway, the Wnt-Fzd-LRP5/6 leads to a downregulation of glycogen synthase kinase-3 (GSK-3) activity that in turn increases β-catenin levels in the cytosol, thereby facilitating its translocation to the nucleus. At the nuclear level, β-catenin forms complexes with members of the Tcf/Lef class of DNA-binding proteins modulating transcriptional activity of target promoters [86,87]. The Wnt-Fzd-LRP5/6 interaction leads to the inhibition of the axin degradasome destruction complex that is recruited to the plasma membrane, helping the interaction between LRP5/6 and axin. When LRP5/6 is phosphorylated at specific amino acidic residues (Ser1490, Thr1530, Thr1572, Ser1590, Ser1607), it acts as a direct competitive inhibitor of GSK3. The Wnt signaling, most probably through LRP5 and LRP6, can also directly activate the mammalian target of rapamycin (mTOR) pathway by decreasing GSK-3-mediated activation of the TSC2/TSC1 complex. The activation of mTOR signaling promotes cell growth in addition to representing a vital regulator of autophagy [88].

LRP5 and LRP6 receptors are expressed differently in various tissues and organs. In humans, LRP5 expression level is the highest in liver, while substantial level of expression is also observed in pancreas, prostate, placenta and small intestine. The expression level of this receptor in brain and peripheral leukocytes is very low [89]. Human LRP6 expression is highest in the ovary, with significant levels in the heart, brain, placenta, lung, kidney, pancreas, spleen and testis. The expression of LRP6 is very low in peripheral blood leukocytes, thymus and small intestine [81].

LRP5 and LRP6 have been shown to play important roles in a broad panel of cancers. This is supported by the fact that the main pathway activated by both receptors is the Wnt/β-catenin pathway, which represents a pleiotropic signal involved in cell growth, cell proliferation and polarity, cell differentiation and tissue development [90,91] with a potential link to several diseases including cancer [92,93,94].

LRP5 has been reported to be involved in mediating Wnt/β-catenin signaling in skeletal metastasis prostate cancer (PC) due to the increased levels of Wnt-1 and β-catenin proteins in both PC cell lines and primary specimens [95]. The presence of LRP5 has also been linked significantly with tumor metastasis such as the chondroblastic subtype of osteosarcoma [96].

LRP6 overexpression, observed in many types of cancer and malignant tissues, leads to an abnormal Wnt pathway that is linked to tumorigenesis [97]. In humans, some LRP6 single-nucleotide polymorphisms (SNPs) and mutations in the LRP6 gene have been associated with increased or decreased risk of cancer development [98]. For example, an LRP6 variant (rs6488507) in non-small-cell lung cancer (NSCLC) patients is linked to an increase in the risk of NSCLC in tobacco smokers [99]. Moreover, the overexpression of LRP6 has been documented in breast cancer [97], hepatocellular carcinoma [100], colorectal cancer [101] and pancreatic ductal adenocarcinoma (PDAC) KRAS-dependent pancreatic cancer [102]. Consistent with these observations, a reduction of LRP6 expression and/or activity inhibits cancer cell proliferation and delays tumor growth in vivo [98,103]. Although mainly known as a co-receptor of the Wnt signaling pathway and therefore related to proliferative cellular pathways, some studies have described a connection of LRP6 to neurodegeneration. The LRP6-mediated Wnt signaling pathway is, in fact, compromised in AD brains, and its deficiency in an AD mouse model exacerbates amyloid pathology, synapse loss and Aβ toxicity, synergistically accelerating AD progression [104].

### 2.8. LRP1B

LRP1B is closely related to LRP1 due to their overlapping structural features and thus have shared ligands such as complexes of urokinase plasminogen activator, plasminogen activator inhibitor type-1 and receptor-associated protein (RAP) [105]. Both uPA and PAI-1 are key components of the uPA system, one of the major extracellular matrix-degrading proteinase systems playing a central role in cancer invasion and metastasis as well as other physiological and pathological processes involved in tissue remodeling [106]. LRP1B ligands link the receptor to altered cellular invasion/metastasis and support the correlations observed between LRP1B genetic and epigenetic alterations and many types of cancer [107,108,109], including urothelial malignancies [110], esophageal squamous cell carcinomas [111], gliomas, cervical adenocarcinomas, B-cell lymphomas [112], leukemias [113] and primary pulmonary adenocarcinomas [114]. In particular, its inactivation in 40% of NSCLC cell lines caused this receptor to initially be named LRP-deleted in tumors (LRP-DIT) [115]. Despite its structural link to LRP1, however, LRP1B differs from LRP in terms of tissue expression, localizing mainly in the brain (cortex, hippocampus and cerebellum in neurons, activated astrocytes and microglia), thyroid and salivary gland [105,116,117]. Its wide expression in the brain may suggest a yet-unclear CNS-related biological function, in part supported by its link to Aβ generation. In fact, Cam et al., have shown that LRP1B decreases APP internalization, thus reducing Aβ generation in a framework of anti-amyloid activity [118].

**Table 1:** CNS/neurodegeneration and the cancer-related roles of the LDL receptors. The table summarizes roles and relative molecular pathways of all LDLR (except LRP8, which is discussed later in detail) in CNS/neurodegeneneration and cancer.

LDLR Family Members	CNS Roles/ Neurodegeneration	Molecular Pathway	Refs.	Cancer Related Roles	Molecular Pathway	Refs.
LDLR	Modulation of amyloid clearance and/or deposition	ApoE interaction	[25,27,28]	Downregulation of cell proliferation and metastasis	Downregulation of LDLR through MEK/ERK stimulation	[29]
LRP1	Neuronal survival	ApoE-dependent activation of PKCδ and inactivation of GSK3β	[35]			
	APP trafficking regulation and processing and Aβ clearance	LRP1 antagonist RAP increases cell surface levels of APP and significantly reduce Aβ synthesis. In the absence of LRP1, Aβ production, APP secretion, APP internalization, turnover of full-length APP and stability of APP C-terminal fragments are affected. At the site of the BBB, surface LRP1-mediated extrusion of cerebral Aβ into the luminal side Soluble LRP1 in the periphery sequesters free Aβ in circulation.	[36,37,38,39]	Cellular migration and invasion	Expression of MMP-2 and MMP-9 through ERK in human glioblastoma Serpin PN-1-dependend MMP-9 expression through ERK activation in breast cancer	[43,44]
	Calcium-related cellular processes	ApoE4, but not ApoE3, significantly increased the resting calcium, the calcium response to NMDA-R and the neurotoxicity.	[34]	Cell proliferation, tumor invasion and angiogenesis	LRP1 expression has been linked to neoplastic aggressiveness due to high histological grade and elevated mitotic index. Regeneration of the uPAR receptor system	[45]
	Neurite outgrowth, synaptic plasticity, learning and memory modulation	Upon TTR binding to LRP2, Src, NMDA-Rs, ERK1/2, CREB and Akt activation and/or a pathway involving RIP and the formation of LRP2-ICD MT-IIA binding to LRP2 stimulates neurite outgrowth via signal transduction pathways activated by the NPxY motifs of LRP2.	[49,52]	Cell survival and proliferation	LRP2 is frequently expressed in malignant melanoma. Modulation of phosphorylated Akt and ERK levels	[7]
VLDLR	Regulation of the migration and layering of the neurons in the cortex and the cerebellum	Reelin-induced Dab1 binding to VLDLR activates SFK and Abl families, together with LRP8. Pafah1b complex mediates downstream effects of VLDLR on neuronal migration.	[59,60,119]	Cell proliferation, migration and metastasis	VLDLR II is overexpressed in lymph node and distant metastasis in gastric and breast cancer patients, promoting cell proliferation and migration. ATRA attenuates proliferation and migration through significant decreases in VLDLR II, while PMA has the opposite effect on VLDLR II, which activates β-catenin/TCF signaling and modulation of MMP-2 and MMP-9.	[8,61]
LRP4	Synaptic homeostasis	LRP4 mutant astrocytes suppressed glutamatergic transmission by enhancing the release of ATP.	[63]			
	Synaptic transmission, LTP and cognitive function	LRP4 KO shows deficits in cognitive tasks with aberrant synapse form and function and loss of LTP.	[65]	EMT promotion	LRP4 is overexpressed in papillary thyroid and gastric cancers, where it promotes EMT through PI3K/AKT pathway and modulation of N-cadherin, ZEB1 and EZH2.	[70,71]
	Adult hippocampal neurogenesis	LRP4 mutation blocks NPSC proliferation. Agrin-LRP4-Ror2 signaling is involved in NSPC proliferation.	[66]	Cell proliferation, migration and invasion	LRP8 downregulation affects colony formation and migratory and invasive capacities through PI3K/AKT pathway. miR-140-5p negatively regulates LRP4.	[70,71]
	Formation and maintenance of the neuromuscular junction	Agrin–LRP4 interaction via MuSK, Dok7 and rapsyn mediates AChR clustering.	[67,68]			
LR11/ SorLA	Regulation of APP processing and Aβ levels	Interaction with APP, enhancement of APP in endosomal compartments and Golgi, modulation of APP processing and reduction of Aβ levels	[72,73]	Cell proliferation	Regulation of endosomal trafficking and oncogenic fitness of HER2, promoting PI3K-dependent HER2 signaling	[78]
LRP6	Synaptic function and integrity	Activation of Wnt signaling	[104]	Cell proliferation, survival and differentiation, tumor growth	Co-receptor for WNT and Wnt activator	[98,103]
LRP1B	Regulation of APP endocytic rate and Aβ levels reduction	Interaction with APP	[118]	Suppression of cell growth, invasion, migration, colony and tumor formation	Reduction of matrix metalloproteinase 2 level and negative regulation of uPAR DNA methylation	[106,107,108]

Aβ, amyloid beta; BBB, blood-brain barrier; MMP-2, matrix metalloproteinase-2; MMP-9, matrix metalloproteinase-9; NMDA-R, N-methyl-D-aspartate receptor; uPAR, urokinase-type plasminogen activator receptor; MAP, mitogen-activated protein; CREB, cAMP response element-binding protein; MT, metallothionein; RIP, regulated intramembrane proteolysis; ICD, intracellular domain; SFK, Src kinase family; ATRA, All-trans retinoic acid; PMA, phorbol-12-myristate-13-acetate; LTP, long term potentiation; EMT, epithelial-mesenchymal transition; NPSCs, neural progenitor stem cells; AChR, acetylcholine receptor; PI3K, phosphoinositide 3-kinases; MuSK, muscle-specific Tyr kinase.

## 3. Apolipoprotein E Receptor 2 (LRP8)

**Figure 2:**
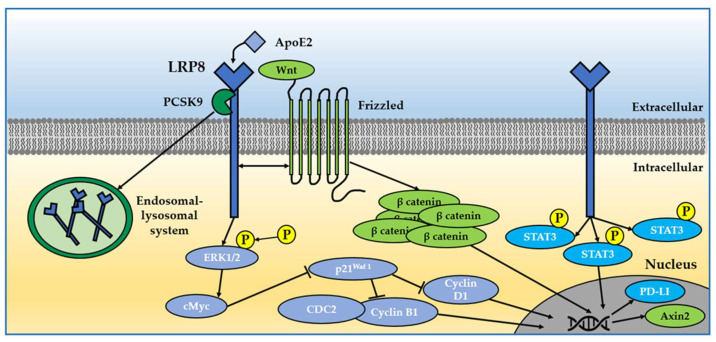
LRP8 in four main cancer-related molecular pathways. (1) LRP8 binding to ApoE2 induces ERK1/2 phosphorylation and cell cycle activation [150]. (2) LRP8 promotes Wnt-induced β-catenin accumulation, inducing Axin2 transcription [151]. (3) PCSK9 modulates LRP8 endocytosis and signaling by targeting the receptor for lysosomal degradation [152]. (4) LRP8 activates p-STAT3 and its nuclear signaling [141,153].

**Figure 3:**
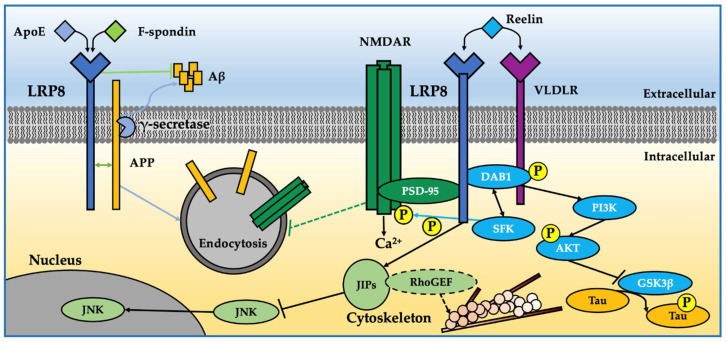
LRP8 in CNS related molecular pathways and neurodegeneration. Ligands binding to LRP8 modulate intracellular pathways, LRP8 cleavage, LRP8-APP interaction and Aβ production. (1) ApoE. Aβ production is increased by ApoE binding to LRP8 [157]. (2) F-Spondin. F-Spondin increases LRP8 cleavage and decreases Aβ production [158]. (3) LRP8 levels also modulate Aβ production in a positive manner by increasing the APP association with lipid rafts and γ-secretase activity [159]. (4) Reelin. Reelin binding to LRP8 induces different signaling pathways: (a) the tyrosin phosphorylation of Dab1 by Src-family kinases [160] and its interaction with the LRP8 NPxY motif. Phosphorylated Dab1 subsequently interacts with PI3K, whose activation leads to the further activation of Akt, which in turn inhibits GSK3β, suppressing tau phosphorylation [161]; (b) the LRP8-JIPS interaction at the plasma membrane level that interferes in turn with the signaling of JNK, whose translocation into the nucleus is therefore inhibited. In addition, the activity of rhoGEF, which is associated with JIP, might be modulated by the Reelin-LTP8-JIPs complex [18,162]; (c) the promotion of LRP8, Dab1 and NMDA-R clustering and related NMDA-R phosphorylation mediated by SFK, which in turn leads to an increase in calcium influx [163] (Chen, 2005). The signal activated by LRP8 and NMDA-R complex, but not necessarily their physical association, involves PSD-95, which is known to inhibit NMDA-R internalization [164].

LRP8 (gene name *LRP8)* is a modular type I transmembrane receptor of the LDLR family whose structure is made of one N-terminal extracellular ligand-binding domain made of seven conserved LA repeats, an EGF domain made of three EGF repeats and one β-propeller and one NPxY motif in the intracellular domain, as described in Figure 1 [120]. LRP8 is involved in many intracellular signaling pathways and cellular responses involving different ligands and co-receptors that will be discussed in detail in Section 3.1 and Section 3.2 and in the respective Figure 2 and Figure 3. The intracellular signaling pathways mediated by LRP8 mostly involve the carboxy-terminal NPXY motif [121,122]. This motif adopts in fact a tight hairpin conformation required for binding to phospho-tyrosine binding (PTB) domains or to SH2-SH3 domain-containing proteins. Ligand binding to LRP8, together with different LRP8 spliced variants, also modulates its proteolytic processing that releases C-terminal fragments (CTF) and a transcriptionally active intracellular domain (ICD) [123,124]. A detailed molecular mechanism of LRP8 proteolitic processing is described in Section 3.2.

As mentioned above, LRP8 has several splice variants, expressed in a tissue-specific manner [125,126,127], whose alteration has also been correlated to sporadic Alzheimer’s disease [128], that modify LRP8 functions such as ligand-binding activity [129], receptor glycosylation and processing [130] and downstream signaling [131]. A well-studied LRP8-spliced variant is the LR7/8B, which harbors high ligand-binding repeats and is known to be a receptor for α-macroglobulin together with LRP1 [132]. Another spliced variant, reported in all placental mammals so far studied and not in marsupials, birds or reptiles, includes exon 19 that encodes for a 59 amino acid proline-rich insert in the cytoplasmic domain of LRP8, which is unique in the LDLR family [133,134]. The human LRP8 unique aminoacidic sequence contains two potential SH3 binding motifs, PXXP (two prolines spaced by two other amino acids), suggesting a role in signal transduction pathways involving reelin, PSD-95, NMDA-R and JIPs and the corresponding functional roles in memory and spatial learning [16,18]. Indeed, its deletion in the mouse *LRP8* gene (exon 19) explains defects in long-term memory storage and spatial learning, most probably due to the interruption of the reelin-mediated activation of NMDA receptors [134].

Additionally, there are variants missing epidermal growth factor repeat B or the O-glycosylation domain [126]. It has been reported that differential LRP8 splicing, together with its glycosylation at the O-linked sugar, regulates the release of the ICD. In this context, Wasser et al., have reported an alternative LRP8 splice variant lacking the O-linked glycosylation region that is cleaved more efficiently than the full-length counterparts [130]. More recently, it has been shown that LRP8 isoforms with differing numbers of ligand-binding repeats to generate different amounts of CTFs compared with full-length LRP8 [135].

LRP8 is enriched in the brain, in particular in the neocortex, cerebellum, hippocampus and olfactory bulb [136], and to a minor extent also in the peripheral nervous system such as in the sciatic nerve and Schwann cells [137]. Outside the nervous system, LRP8 can be found in placenta, testis, ovary and platelets and also in immune cells and vascular endothelial and smooth muscle cells [120,138]. Unlike LRP1, LRP8 inactivation has no impact on plasma triglyceride or cholesterol levels. Hence, the association between polymorphisms in the LRP8 gene and CVD risk is likely independent of lipid metabolism but related to LRP8-modulating cellular functions in the vessel wall [139].

### 3.1. LRP8 and Cancer

Although, LRP8 is mainly recognized for its role in CNS-related pathways, scientific evidence also suggests an involvement of the receptor in carcinogenesis. Generally, cancer cells require higher uptake of cholesterol than normal cells in which the receptor-mediated endocytosis of serum LDL enhances the cholesterol content through LDLR. Increases in LDLR and elevated plasma low-density lipoprotein cholesterol (LDL-C) have been reported as features of leukemia, glioblastoma and lung and pancreatic tumors [140]. This is explained by the fact that tumor cells rely on cholesterol for membrane and lipid raft biosynthesis, signaling molecules and other factors in order to meet the fast growth.

In this context, many studies summarized in Table 2 have highlighted the role of LRP8 in the tumorigenesis and progression of several cancers such as osteosarcoma, breast cancer, gastric cancer, hepatocellular carcinoma, lung cancer, prostate cancer and pancreatic cancer [6].

In fact, LRP8 is overexpressed in different cancers including osteosarcoma [141] and NSCLC [148], and its overexpression has been significantly correlated with poor clinicopathological features and prognosis. Abnormal LRP8 expression has also been associated with breast cancer progression, where it facilitates cell growth and confers a poor prognosis in patients. In particular, Maire et al., demonstrated that LRP8 is more strongly expressed in breast cancer without hormone receptor expression (TNBC and HER2 positive) than in luminal tumors (Luminal A and Luminal B) and that LRP8 depletion promotes apoptosis and impaired cell proliferation and colony formation. These findings have been further confirmed in an in vivo xenograft model where LRP8 depletion slowed tumor growth [142]. Other studies indirectly support the idea of a role of LRP8 in tumorigenesis: Dun et al., have shown that mycophenolic acid severely downregulates the expression of cell surface LRP8, inhibiting cell migration and the invasion of gastric cancer cells [143]; Cai et al., have separately found that LRP8 is responsible for hepatocellular carcinoma cell resistance to sorafenib [145].

In a scenario in which LRP8 seems to have an oncogenic role in many types of cancer, it remains still elusive the biochemical pathways through which LRP8 exerts such activity (Figure 2).

One hypothetical mechanism is linked to ApoE isoform 2, which is known to have a critical role in cell proliferation and has been identified as a cancer-related molecule. Through its binding to LRP8, ApoE2 induces ERK1/2 phosphorylation, activating c-Myc that in turn inhibits p21^Waf1^ and thus removing the brake on the expression of cycle-related proteins such as cyclin D1, cdc2 and cyclin B1 [150].

In osteosarcoma, the overexpression of LRP8 compared with normal tissues, increases phospho-STAT3 (p-STAT3) levels and facilitates its translocation into the nucleus, resulting in an enhancement of mRNA, protein and promoter activity of the programmed death-ligand 1 (PD-L1), which is a marker of metastasis and mortality risk in osteosarcoma [141].

Among the intracellular signaling pathways positively regulated by LRP8, the Wnt/β-catenin is definitely one that links LRP8 to its tumorigenic role [151]. As already reported above, the Wnt/β-catenin pathway is a pleiotropic signal linked to several diseases including cancer. In particular, LRP8 seems to be a positive regulator of the Wnt pathway, increasing Wnt-induced transcriptional responses and promoting Wnt-induced β-catenin accumulation. The LRP8 regulation of the Wnt/β-Catenin pathway has been observed both in triple-negative breast cancer, where LRP8 depletion inhibits breast cancer stem cells, cell proliferation and invasion and epithelial–mesenchymal transition [9], and in NSLC, in which an increase in LRP8 expression facilitates tumor proliferation [148]. Furthermore, in KS483 osteoprogenitor cells, knockdown of LRP8 results in a decrease in the Wnt/β-Catenin pathway, with reduced β-catenin levels and the suppression of Axin2 transcription; as a secondary effect, the depletion of LRP8 decreases osteoblast differentiation and mineralization, whereas LRP8 ectopic expression has the opposite effect [151].

Together with VLDLR and LRP1, LRP8 is also involved in tumor cell growth through its binding to the glycoprotein Proprotein convertase subtilisin/kexin type 9 (PCSK9). PCSK9 is known to modulate cholesterol metabolism through its binding to LDLR family members, including LRP8, and promoting their degradation in intracellular acidic compartments. The binding between PCSK9 and the LDLR members occurs between the PCSK9 catalytic subunit and the LDLR EGF-A domain [152]. In this context, in addition to being a key player in the LDL metabolism in the liver and in the brain, PCSK9 is overexpressed in various human cancer cell lines [154].

Komaravolu et al., reported a cell-cycle modulation, not strictly related to cancer, that was mediated by LRP8 through its intracellular interaction with the catalytic subunit of the heterotrimeric enzyme PP2A, a protein complex that is vitally important for the maintenance of normal cell division [155,156]. In this regard, LRP8 participates in mitosis via its interaction with PP2A-C, promoting the formation of active CDC20 to complete cytokinesis. Moreover, since the LRP8-interacting adaptor protein Dab2 is also a substrate for PP2A dephosphorylation, LRP8 may work in concert with Dab2 to modulate cell cycle progression and cytokinesis [156].

LRP8 is also a key mediator in metastatic melanoma, as an ApoE receptor, in parallel with VLDLR and LRP1. According to Pencheva et al., selected miRNAs (miR-1908, miR-199a-5p and miR-199a-3p) limit ApoE secretion, thus suppressing its anti-metastatic action through the engagement of endothelial LRP8 receptor; in this condition, LRP8 no longer suppresses cell invasion and endothelial recruitment, eventually promoting metastasis [146].

Consistent with the above observations, LRP8 was also identified as a target of miR-30b-5p in lung cancer progression and cisplatin resistance. In particular, LRP8 level was markedly increased in lung cancer tissues compared with the control group, apparently acting as an oncogene. In addition, miR-30b-5p inhibits lung cancer cell viability, migration and invasion and enhances cell sensitivity to cysplatin via targeting LRP8, suggesting that miR-30b-5p inhibits lung cancer progression by targeting LRP8 [147]. Lu et al., published a similar work highlighting the role of miR-142 as a gastric cancer suppressor by targeting LRP8 [144]. A similar LRP8 regulation by miR-455-5p has been reported by Arai at al. in prostate cancer [149].

### 3.2. LRP8 in Neurodegeneration

LRP8 has long been studied for its role in cholesterol transport and metabolism; however, the identification of ApoE4, the major genetic risk factor for developing late-onset Alzheimer’s disease, as one of its ligands has brought attention to its role in CNS-related pathways and neurodegeneration (Figure 3).

LRP8 is mainly known for being a receptor of ApoE, which is produced in various organs, including liver, brain, kidneys, and adrenal glands, and which represents a component of lipoproteins responsible for packaging cholesterol and other fats and carrying them through the bloodstream [165]. LRP8 binds with high affinity only to ApoE-rich β-VLDL, while its affinity for LDL and other VLDL is much lower [120]. In humans, three ApoE isoforms exist that differ at positions 112 and 158 (ApoE2, ApoE3 and ApoE4) [166]. ApoE2 has cysteines at positions 112 and 158, ApoE3 has a cysteine at position 112 and an arginine at position 158 and ApoE4 has arginines at positions 112 and 158. The ApoE ε2 allele is associated with the recessive inheritance of hyperlipoproteinemia in patients with type III hyperlipidemia [167], and the ε4 allele is strongly associated with cardio-cerebrovascular diseases and Alzheimer’s disease [168]. A single copy of the ε4 allele increases AD risk by about 3-fold, with two copies of the ε4 allele increasing disease risk about 12-fold relative to individuals who have 2 copies of the ε3 allele. On the other side, the ε2 allele seems to decrease AD risk by about 0.6 [169,170]. Additional studies have demonstrated that ApoE is closely related to cognitive functions; indeed, ApoE-deficient homozygous mice show impaired working memory [171] accompanied by the age-dependent loss of synaptic proteins [172]. In addition to AD, ApoE has also been reported to have an impact on dementia and synucleinopathy in Parkinson’s disease (PD) dementia and dementia with Lewy bodies as well as in mouse models of synucleinopathy [173,174]. ApoE is also directly involved in the binding and clearance of Aβ, with ApoE ε3 and ApoE ε2 having a higher affinity compared with ApoE ε4 [175,176]. Moreover, these data were also confirmed in in vivo studies showing that knockout ApoE mice crossed with AD mice overexpressing APP exhibit less Aβ deposition compared with those expressing ApoE ε3 or ApoE ε4 [177,178].

In Alzheimer’s disease, Aβ is generated from APP through sequential cleavages, first by β-secretase and then by γ-secretase complex, the latter consisting of at least four components: presenilin (PS, PS1 or PS2), Nicastrin, anterior pharynx-defective-1 (APH-1) and presenilin enhancer-2 (PEN-2) [179,180]. PSs are the crucial catalytic components of γ-secretase, and mutations in PS1 are causative in the majority of familial AD (FAD) cases, likely through a yet-unclear loss of function, while APP overexpression or mutations are causative of FAD, likely favoring amyloidosis and tauopathy [181,182].

Hoe et al., uncovered interactions between LRP8, APP and F-spondin, a component of the extracellular matrix involved in neuronal migration and plasticity in adult and developing brains, suggesting that interaction between extracellular matrix proteins and LRP8 at the cell surface can alter APP processing and decrease Aβ production. LRP8 and APP interaction happens at the extracellular and intracellular levels and increases with the presence of F-spondin, which also mediates increased LRP8 cleavage [158].

The potential clustering of APP and LRP8 modulates their endocytosis, likely altering the cleavage of each receptor, via both extracellular metalloproteinases and intramembranous γ-secretase cleavage. Interestingly, LRP8 expression also correlates with a significant increase in Aβ production and reduced levels of APP-CTFs. The increased Aβ production seems, in turn, to depend on the integrity of the NPXY endocytosis motif of LRP8; in fact, LRP8 expression increases APP association with lipid rafts and γ-secretase activity, both of which might contribute to increased Aβ production [159]. Moreover, ApoE binding to LRP8 also increases APP endocytosis and Aβ production in an isoform-specific manner [157]. There is therefore a link between ApoE and Aβ that does not depend on their direct interaction but rather on the ability of the different isoforms of ApoE to modify the processing of APP through LRP8, thus representing a bidirectional bridge between cholesterol metabolism and amyloidosis.

In addition to ApoE, another well-known ligand of LRP8 is Reelin: which is ligand as well of the VLDL receptor and that is produced by Cajal–Retzius neurons at the surface of the developing neocortex [183]. Depending on the specific tissue and cell type involved, the binding of Reelin to LRP8 leads to the tyrosine phosphorylation of the adaptor protein Dab1/2 to initiate several signaling cascades such as the activation of PI3K, ERK1/2, Src-family kinases and protein kinase B/Akt. In this context, it is important to highlight that Dab1 is essential for neuronal migration both upon LRP8 or APP activation, suggesting that it is likely through Dab1 that both APP and LRP8 regulate neuronal migration from SVZ toward the cortical plate [59,184]. Furthermore, Stockinger et al., have reported a role of the LRP8-Reelin interaction in the correct positioning of neurons during the embryonic development of the brain.

In the CNS, Reelin is able to regulate APP processing and Aβ production by interacting with APP. Reelin increases cell surface levels of APP and decreasedthe endocytosis of APP in hippocampal neurons in vitro. In vivo, Reelin levels were increased in the brains of APP knockout mice and decreased in APP-overexpressing mice [185].

Reelin signaling is crucial for many CNS functions such as neuronal migration, dendritic spine development [186], synaptic plasticity [184,187,188] and brain embryonic development [59]. Some of the Reelin functions are played through binding to LRP8; indeed, LRP8 Reelin-regulated neuronal (LRN) enhancer binding modulates learning and memory, which activates synaptic plasticity genes including NMDA receptor NR1, NR2A and NR2B [124]. In addition to its NMDA gene activation, Reelin signaling through LRP8 induces the tyrosine phosphorylation of the receptor NMDA (NR2) subunits, changing their surface distribution and activity and in turn regulating synaptic plasticity, which is further modulated by the differential splicing of the LRP8 cytoplasmic domain [188]. Moreover, LRP8 proline rich 59 amino acid insert is essential for the Reelin-induced enhancement of LTP in mice [59]; in this context, interference with Reelin expression or its intracellular signaling pathway has effects on LTP and seizure potential that are partially replicated in LRP8 knockouts. In this context, presenilin mutations have also been linked to Reelin-signaling elements such as phosphatidylinositide 3-kinase and Crk/Dock1/Rac, highlighting a potential correlation of presenilin mutations on synaptic processes with LRP8 [189,190].

Additional studies on LRP8-KO mice have supported a role of LRP8 in neuronal functions, showing in particular a severe impairment in freezing behaviors by performing a classical form of robust associative learning (contextual fear paradigm) reflecting a decline in long-term memory formation [124]. Moreover, LRP8 deficiency in mice has been shown to affect the development of the neocortex, preventing neurons from completing their migration and causing in turn a partial inversion of the neuronal layers in the neocortex [59]. In this context, LRP8 KO/ApoE KO mice have shown cognitive decline greater than that observed in ApoE single-KO mice as indicated by behavioral tests [191].

In addition to the activation of intracellular signaling pathways, the binding of some ligands, especially Reelin, to LRP8 also regulates its proteolytic processing. From the LRP8 full-length mature form (105 kDa), two major proteolytic products have been described so far [88,166,167,168], the carboxy-terminal fragment generated by extracellular α-secretase (20–25 kDa) and a C-terminal fragment (15–18 kDa) that is produced by γ-secretase cleavage and is similar to AICDs derived by APP processing [166]. In fact, LRP8 is a γ-secretase substrate, like APP and other LDL members such as LRP1 [169], LRP1b [170], LRP2 [171], LRP6 [172], LR11 [173] and VLDLR [174] as well as many other substrates including Notch [175,176,177,178]. The LRP8 C-terminal fragment produced by γ-secretase cleavage appears to translocate into the nucleus where it inhibits Reelin transcription, creating a negative feedback loop for Reelin levels; indeed, depletion of Presenilin1, the catalytic component of γ-secretase, elevates the levels of both Reelin and LRP8 in mouse brain [123]. Telese et al., showed that the nuclear translocation of the LRP8 CTF, derived from γ-secretase cut, stimulates specific enhancers critically involved in hippocampus-dependent learning and memory, implying that Reelin can impact genes related to learning and memory via the LRP8 γ-CTF and that this process might be attenuated by PS1 mutations [124]. In this scenario, in which the processing of LRP8 modulates its functions, Reelin causes a reduction of the full-length receptor, probably enhancing its proteolysis by a sheddase after ligand-stimulated internalization. PS1 mutations show impaired γ-secretase activity relatively to LRP8 and an accumulation of its C-terminal fragments after Reelin exposure; this last effect is potentiated by co-exposure to DAPT, a γ-secretase inhibitor [192].

In addition to the ligands already mentioned, ApoE, F-Spondin and Reelin, other proposed LRP8 ligands are: (1) trombospondin-1, expressed in the subventricular zone and throughout the rostral migratory stream, where it acts on LRP8 by promoting neuroblast chain migration [193]; (2) clusterin (apolipoprotein J), which modulates a cell proliferative signal in migrating neuronal precursors of SVZ explants, in vitro, via Dab1 activation [194]; (3) selenoprotein P, a mediator of selenium transport whose binding with LRP8 has been observed in the brain, testis and bone. Selenium and its carrier protein, selenoprotein P, have been reported to have important roles in maintaining optimal brain function as well as in relevant AD-related pathways in addition to regulating the development and the immune system and having antitumor properties due to their strong antioxidant activities [195]. Epidemiological studies have shown a significant positive correlation between selenium level and cognitive ability [196] and that blood selenium level gradually decreases with age [197]. Furthermore, selenium levels change significantly in the brain and blood of patients with various neurodegenerative diseases such as AD, PD, multiple sclerosis and Batten’s disease [198]. In this context, LRP8 KO mice have shown similar phenotypes of selenoprotein P KO mice in the brain and in the testis, highlighting the biological significance of the receptor-mediated uptake of selenium. Indeed, lowering brain Se levels by the genetic inactivation of selenoprotein P, or its neuronal receptor LRP8 [199,200], has been shown to lead to spontaneous neurological deficits and neurodegeneration.

An overview of the LRP8 roles and molecular mechanisms in CNS and neurodegeneration is reported in Table 3.

## 4. Final Remarks

LDLR, and LRP8 in particular, are receptors that through multiple pathways and mechanisms seems to play a role both in the modulation of neuronal activity and in the regulation of cell proliferation. In this context, several studies have proposed a potential link between oncological and neurodegenerative pathways but have rarely found a common denominator. Using an obvious oversimplification, we could say that high levels of LRP8 manifest a correlation with proliferation and metastasis in cancer cells and tissues, while functions related to neuronal migration, amyloidosis and neurodegeneration, in a broad sense, are likely modulated by the proteolytic processing of LRP8, which however has never been fully defined in its complexity.

Some features of LRP8 are, from this point of view, relevant: from one side, the same pathways (for example Wnt) foresee LRP8 as pivotal regulator both in degeneration like Alzheimer’s and in tumor genesis such as in breast cancer; from the other side, ApoE, the most famous ligand of LRP8, is at the crossroads between metastatic proliferation and Alzheimer’s-type neurodegeneration. At present, it is not clear what is the main pathway through which LRP8 can influence tumor proliferation or whether there is a prevalent mechanism in which it is involved. Specific studies on cancer stem cells and using in vivo xenograft animal models will need to clarify these aspects before LRP8 can be defined as a potential therapeutic marker.

In addition, it is well-known that the processing of many receptors involved in oncological and/or neurodegenerative pathways depends on the action of the intramembrane protease γ-secretase. In fact, γ-secretase inhibitors are still used in oncological trials and in the recent past have been used, with unfortunately negative results, to treat patients suffering from Alzheimer’s disease. Regarding LRP8, it is not known if its processing by γ-secretase is altered in patients with Alzheimer’s disease, and the consequences of this processing on amyloid formation are still underexplored.

After putting together the pieces of the puzzle, there are still obvious questions that remain open and whose answers could lead not only to decipher key points in the intersection between oncological and neurodegenerative pathways but also to define new therapeutic targets in both fields: One, what is the exact role of ApoE/LRP8 in tumorigenesis and Alzheimer’s? Two, how important are the different isoforms of splicing and the complex processing of LRP8 to defining its functions in AD and cancer? Three, can amyloidosis and cell proliferation in cancer be reversed targeting ApoE/LRP8? In this context, it is sadly remarkable that the hypothesis of a loss of function of γ-secretase in familial AD mutants, relative to other substrates than APP, has never been studied in depth [201,202]. The answers to these questions will hopefully lead to a greater knowledge of the molecular mechanisms in which LDLR receptors are involved, and LRP8 in particular, to develop new diagnostic and therapeutic tools that can be used both in oncology and in some neurodegenerative conditions such as the Alzheimer’s dementia and pathologies related to aberrant neuronal migration.

## Figures and Tables

**Table 2:** LRP8 in cancer-related molecular pathways. The table summarizes LRP8 main findings in cancer, with the corresponding roles, molecular mechanisms and in vitro and in vivo models used (see references).

Cancer Types	LRP8 Cancer Roles	Main Findings & Molecular Mechanism	In Vitro E in Vivo Models	Refs.
Osteosarcoma	Cell proliferation and anti-apoptotic effect	LRP8 is overexpressed in osteosarcoma tissues. LRP8 enhances PD-L1 expression via STAT3, evading the host immune system.	Cell lines: MG63 and U2OS	[141]
TNBC TNBC	Cell proliferation, anti-apoptotic effect and colony formation	LRP8 is overexpressed in TNBC patients. LRP8 depletion induces arrest of the cell cycle and apoptosis. LRP8 knockdown impairs colony formation.	Cell lines: BT-474, T47D, MCF7, ZR-75-1, SKBR3, HCC1569, HCC1954, BT-20, HCC1143, HCC38, HCC70, MDA-MB-468 and MDA-MB-45 In vivo model: xenograft mice model (MDA-MB-468)	[142]
	Tumorigenesis and chemoresistance	LRP8 silencing suppresses BCSCs and tumorigenesis in TNBC via Wnt signaling inhibition. LRP8 KO shifts TNBC cells to a more differentiated phenotype, sensitizing them to chemotherapy.	Cell lines: HCC1937 and SUM149 In vivo model: xenograft NOD/SCID mice (SUM149)	[9]
Gastric cancer	Cell migration	Mycophenolic acid downregulates LRP8, reducing cell migration.	Cell lines: AGS and Hs746T	[143]
	Cancer progression	MiR-142 suppresses progression of gastric carcinoma via directly targeting LRP8.	Cell lines: AGS, MKN-45, MKN-28, SGC-7901 and BGC-823	[144]
Hepatocellular carcinoma	Pharmacoresistance	LRP8-dependent activation of β-catenin pathway suppresses Sorafenib induced apoptosis.	Cell lines: Huh7 and MHCC-97H	[145]
Melanoma	Suppression of cell invasion and endothelial recruitment	miR-1908, miR-199a-5p, and miR-199a-3p limit ApoE secretion suppressing LRP8 endothelial engagement	Cell lines: TWM-266-4, A375, SK-Mel-28, HT-144, A2058, MeWo, SK-Mel-2, SK-Mel-28, A375, WM-266-4, HT-144, and A2058 In vivo model: xenograft NOD scid mice (MeWo)	[146]
Lung cancer	Cancer progression and cisplatin resistance	miR-30b-5p inhibits lung cancer cell viability, migration and invasion and enhances cell sensitivity to DDP via targeting LRP8.	Cell lines: A549, A549/DDP, NCI-H1299, NCIH446 and H1650 In vivo model: xenograft BALB/c nude mice	[147]
	Cell proliferation, migration, invasion, EMT, tumor growth (NSCLC)	LRP8 is markedly overexpressed in NSCLC patients with poor clinicopathological characteristics and prognosis. LRP8 KO elicits tumor-suppressive functions by suppressing the Wnt/β-catenin pathway.	Cell lines: 95-D, H1299, H460, HCC-827, A549, PC-9, and H1975	[148]
Prostate cancer	Cancer progression	miR-455-5p inhibits cancer cell migration and invasive abilities through LRP8 downregulation.	Cell lines: PC3, DU145, and C4-2	[149]
Pancreatic cancer	Cell proliferation	ApoE2-LRP8 induces phosphorylation of ERK1/2 to activate c-Myc, promoting cyclin D1, cdc2 and cyclin B1 expression and reducing p21^Waf1^ activity.	Cell lines: MIA PaCa-2, Capan-2, PANC-1, Bxpc-3	[150]

TNBC, Triple-negative breast cancer; BCSCs, Breast cancer stem cells; EMT, Epithelial-to-mesenchymal transition; NSCLC, Non-small cell lung cancer.

**Table 3:** LRP8 in CNS- and neurodegeneration-related molecular pathways. The table summarizes main findings in CNS and neurodegeneration correlating LRP8 and its ligand binding to corresponding roles, molecular mechanisms and in vitro and in vivo models used in the experimental studies taken into consideration (see references).

LRP8 Interactors	Roles in CNS and Neurodegeneration	LRP8 Main Findings & Molecular Mechanism Related to CNS	In Vitro E in Vivo Models	Refs.
ApoE	Neurodegeneration	Increase in APP endocytosis and Aβ production via X11α or X11β (X11α/β)	Neuroblastoma N2a cells	[157]
Reelin	Synaptic plasticity	Activation of synaptic plasticity genes mediated by the activation of neuronal enhancers	Primary cortical neurons/heterozygous Reeler and LRP8-KO mice	[124]
		Enhancement of LRP8 proteolytic processing, followed by LRP8-ICD induced transcription		
		Modulation of NMDA-R phosphorylation via SFKs and Dab1 followed by increased Calcium influx	Primary wild-type cortical neurons/Dab1 knock-out neurons	[163]
	Control of neuronal migration and cellular layer formation in the developing brain	Partial inversion of the neuronal layers in the neocortex	VLDL and LRP8 KO mice	[59]
	Neurodegeneration	Activation of the signaling pathway involving Dab1-PI3K-AKT leading to the inhibition of GSK3β and in turn phosphorylation of tau	Primary neurons	[161]
Trombospondin-1 (THBS-1)	Postnatal neuronal migration	Promotion of neuroblast chain migration	SVZ explants from wild-type mice, ApoER2−/− VLDLR−/− mice and THBS-1−/− mice on a C57BL6/J background	[193]
Clusterin	Postnatal neuronal migration	Modulation of a cell proliferative signal in migrating neuronal precursors via Dab1-PI3K/Akt signal	SVZ explants from wild-type mice	[194]
Selenoprotein P (Sepp1)	Preservation of neurological function and survival	Selenium transport	Sepp1−/− and Sepp1+/+ male mice ApoER2−/− mice (strain name, B6;129S6-Lrp8tm1Her/J)	[199]
F-Spondin	Neurodegeneration	LRP8 cleavage increase and Aβ production decrease	COS7 and HEK293 cells transfected with reelin, spondin, thrombospondin or F-spondin	[158]

SVZ, Subventricular zone.

## Data Availability

Not applicable.
